# PROPER: global protein interaction network alignment through percolation matching

**DOI:** 10.1186/s12859-016-1395-9

**Published:** 2016-12-12

**Authors:** Ehsan Kazemi, Hamed Hassani, Matthias Grossglauser, Hassan Pezeshgi Modarres

**Affiliations:** 1 School of Computer and Communication Sciences, EPFL, Lausanne, Switzerland; 2 Department of Computer Science, ETHZ, Zurich, Switzerland; 3 School of Life Sciences, EPFL, Lausanne, Switzerland

**Keywords:** Global network alignment, Protein-protein interaction, Percolation graph matching, Biological network

## Abstract

**Background:**

The alignment of protein-protein interaction (PPI) networks enables us to uncover the relationships between different species, which leads to a deeper understanding of biological systems. Network alignment can be used to transfer biological knowledge between species. Although different PPI-network alignment algorithms were introduced during the last decade, developing an accurate and scalable algorithm that can find alignments with high biological and structural similarities among PPI networks is still challenging.

**Results:**

In this paper, we introduce a new global network alignment algorithm for PPI networks called PROPER. Compared to other global network alignment methods, our algorithm shows higher accuracy and speed over real PPI datasets and synthetic networks. We show that the PROPER algorithm can detect large portions of conserved biological pathways between species. Also, using a simple parsimonious evolutionary model, we explain why PROPER performs well based on several different comparison criteria.

**Conclusions:**

We highlight that PROPER has high potential in further applications such as detecting biological pathways, finding protein complexes and PPI prediction. The PROPER algorithm is available at http://proper.epfl.ch.

**Electronic supplementary material:**

The online version of this article (doi:10.1186/s12859-016-1395-9) contains supplementary material, which is available to authorized users.

## Background

Proteins are large biomolecules that carry out vital functions in living cells. They typically carry out these functions in concert with other biomolecules, especially other proteins, which enables their diverse functionality, such as forming signaling networks and metabolic pathways, and regulating enzymatic activities [1]. In this context, the term protein-protein interaction (PPI) stands for the mutual interactions between pairs of proteins.

PPI data are obtained by high-throughput experimental techniques such as yeast 2-hybrid [2], synthetic lethality [3] and co-immunoprecipitation coupled mass spectrometry [4]. The data are deposited in more than 100 PPI databases [5] such as BioGRID [6], the Molecular Interaction Database (MINT) [7], the Human Protein Reference Database (HPRD) [8], and IntAct [9]. Despite the large amount of PPI data, the detection of the protein pathways and protein complexes is challenging because many of the PPIs are noisy and non-reproducible.

A comparative analysis of PPI networks provides insight into species evolution and information about evolutionarily conserved biological interactions, such as pathways across multiple species [1, 10–12]. Network alignment (also known as graph matching or network reconciliation in the computer science literature) algorithms were introduced to compare PPI networks between two or more species.

The comparison of PPI networks, by network alignment, shows that there are identical interaction patterns between proteins with high sequence similarity across different species [13]. For example, there are many common protein interactions between proteins in yeast networks and their corresponding protein orthologs in PPI networks of worms [14]. Because functional interactions are often conserved across species and false positives are unlikely to occur in multiple species, network alignment can increase the confidence level of an observed interaction in a database [15].

PPI-network alignment has many applications in areas such as detection of new pathways and of conserved motifs, prediction of the functions of proteins, orthology detection, drug design, protein-protein interaction prediction and phylogenetic tree reconstruction [16, 17].

Generally, PPI-network alignment methods assume that two functional ortholog proteins on two different PPI networks are likely to interact with proteins in the corresponding networks that are functionally orthologs themselves [1, 18, 19]. Following this line of thought, local network alignment (LNA) and global network alignment (GNA) methods are the main approaches for aligning PPI networks [1, 20, 21]. The LNA algorithms search for small but highly conserved subnetworks (e.g., homologous regions of biological pathways or protein complexes) between species, whereas GNA algorithms try to align all (or most of) the proteins to find large subgraphs that are functionally and structurally conserved over all the nodes in the two networks [1, 20, 21].

PPI-network alignment algorithms use topological (e.g., local and global network structures) and biological (e.g., amino acid sequences of proteins) information to align two networks. The topological information is more important than sequence information for aligning functionally conserved interactions [22, 23], hence the focus of network alignment algorithms shifted from using only biological information towards using topological information. Most of the early works on PPI-network alignment, such as PathBLAST [24], NetworkBLAST [10], NetAlign [25], MaWISh [26] and Grælin [27], study the LNA problem. More recent methods, such as IsoRank [28, 29], the GRAAL family [16, 23, 30–32], MAGNA and its successor MAGNA++ [33, 34], SPINAL [35], PINALOG [36], Netcoffee [37] and BEAMS [38], are examples of GNA algorithms.

In this paper, we consider the GNA of only two networks. Singh et al. [28] introduced IsoRank as the first GNA algorithm for PPI networks. The IsoRank algorithm is formulated as an eigenvalue problem, where it first computes a pairwise protein similarity metric (as a convex combination of protein sequence similarities and a structural similarity score), and then generates the final global alignment between the two networks based on this metric. The authors of [39, 40] developed approximation algorithms for efficient computation of the IsoRank similarities. GHOST [41] aligns two networks according to the similarity of spectral signatures of node couples. PINALOG [36] finds the final alignment by matching the communities of the two networks first. The GRAAL (GRAph ALigner) family is a group of GNA methods that use the graphlet-degree signature similarity to align two networks. GRAAL [30] is the first GNA algorithm that uses only structure of the two networks for alignment. It first selects a couple of nodes with high graphlet-degree signature similarity, and then by a seed-and-extend matching procedure it tries to expand the alignment around this couple in a greedy way. In general, a seed-and-extend algorithm starts the alignment procedure from a set of highly similar couples called seed pairs. Then, it proceeds to align iteratively similar couples among neighbors of the seed pairs. H-GRAAL [31] uses the Hungarian algorithm for improving the quality of alignments produced by GRAAL, at the cost of increased computational complexity. To align two networks, MI-GRAAL [16] integrates several metrics such as graphlet-degree signature similarity, local clustering coefficient differences, degree differences and protein sequence similarity. L-GRAAL [23] is the latest algorithm from the GRAAL family; it directly optimizes both the structural and sequence similarities with a heuristic seed-and-extend strategy based on a Lagrangian relaxation. The SPINAL algorithm [35] iteratively grows an alignment based on an a priori computed coarse-grained node similarity score. By using a genetic algorithm, MAGNA [33] tries to optimize the edge conservation between two networks.

In this paper, we design a new global pairwise-network alignment algorithm for PPI networks; it is built upon previous results for graph matching in computer science. We show the excellent performance of our algorithm (in terms of both accuracy and speed) compared to several state-of-the-art algorithms. We also introduce a new measure for evaluating the performance of algorithms in aligning biological pathways between species. We argue the suitability of our algorithm by analyzing its performance in a bigraph-sampling model of network evolution [42–44]. For this random-bigraph model, we use the results of [43, 45] to guarantee the performance of our algorithm.

## Methods

GNA algorithms, by finding a one-to-one mapping of proteins, try to find large conserved sub-networks (as they are indicative of a common ancestor) and network motif^1^ between several species [46]. Global pairwise-network alignment algorithms align proteins of only two species in order to maximize the biological and topological similarities (these concepts are defined precisely later in the text) between aligned proteins; they have been extensively studied in the literature [20, 21, 28, 35, 36].

A PPI network can be represented by a graph *G*(*V*,*E*), where *V* is the set of proteins and each edge (*u*,*v*) in *E* is an indicator of interaction between the two proteins *u* and *v*. Formally speaking, given two networks *G*_1_(*V*_1_,*E*_1_) and *G*_2_(*V*_2_,*E*_2_), the purpose of global network alignment is to identify a bijection between the full (or partial) vertex sets of two networks. The network alignment algorithms use the protein similarities and the network topology. In this study, the pairwise similarities between proteins are computed by the well-known basic local-alignment search tool (BLAST) [47] that considers the alignment of amino acid sequences of those proteins.

### The PROPER algorithm

Network alignment has been an active area of research in the computer science literature. In the context of network analysis, the main goal is to align social graphs from different domains [48, 49]. In computer vision and pattern recognition, graph matching is used to find similar images in a database [50–52].

In many scenarios, there is some side information that could be used in the process of network alignment. For example, it is possible to use information from a small fraction of individuals who elect to reveal their identities in two social networks. Alternatively, some users link their accounts across multiple networks. This set of revealed identities or linked nodes (henceforth called a *seed set*) enables us to initiate an iterative procedure for matching the two graphs. In this regard, one main category of network alignment algorithms, known as *percolation graph matching* (PGM), assumes that there is side information in the form of a seed set of pre-matched node couples. In this class of algorithms, the alignment starts with a small set $\mathcal {A}$ of initial seed couples and percolates to other node couples, i.e., they build the alignment incrementally between nodes of the two networks [45, 48, 53–55].

We use the ideas from PGM algorithms (mainly [45]) to design our PROPER (PROtein-protein interaction network alignment based on PERcolatin) algorithm.

#### PROPER: two steps

In the process of PPI-network alignment by PROPER, initially we have as inputs two PPI networks *G*_1_(*V*_1_,*E*_1_) and *G*_2_(*V*_2_,*E*_2_), the set of pairwise BLAST bit-score similarities (call it $\mathcal {S}$) for couples of proteins in *V*_1_×*V*_2_, and fixed thresholds *ℓ*,*r*>0, where *ℓ* and *r* are the sequence similarity and the local topological similarity thresholds, respectively.

The PROPER algorithm uses the sequence similarities and network structures in a two-stage procedure: (i) At the first step, it uses the sequence similarities to generate a seed set for a PGM algorithm; and (ii) at the second step, to align remaining couples, it uses only the network structure and the seeds generated from the first step as inputs to the PGM algorithm. This is in contrast with many other pairwise-alignment algorithms, where they try to simultaneously maximize a function of both sequence and structural similarities. In this section, we first explain the process of generating seed set $\mathcal {A}$ from $\mathcal {S}$ (the SeedGeneration algorithm). Then, we explain how to align new couples, starting from the set $\mathcal {A}$ (the MapPercolation algorithm).

Initial seeds play an important role in the alignment process. In the PPI setting, the BLAST bit-score is often a good indicator of functional similarities between proteins [56]. In other words, at high levels of sequence similarity it is possible to make a functional inference with an acceptable accuracy [57]. This means that, for couples of proteins with a high sequence similarity it is very likely that they have similar functions. The main approach in this paper is to use such couples as a starting point to find a global alignment. Indeed, the seeds to the PROPER algorithm are those couples of proteins with high sequence similarities. Also, a protein can be aligned with at most one protein from the other species. The degree of similarity between the couples in the seed set $\mathcal {A}$ is controlled by the threshold *ℓ*.

The seed set $\mathcal {A}$ is generated from the pairwise similarities (the set $\mathcal {S}$) in the following manner: Among all the couples of proteins with BLAST bit-score similarity above *ℓ*, couples [ *i*,*j*] are matched in a descending order of sequence similarity, unless *i* or *j* is matched already. More precisely, (i) we add the couple $[\!i,j] \in \mathcal {S}$ with the highest similarity to the seed set and match *i* to *j*; (ii) all the couples [ *i*,*j* ^′^] and [ *i* ^′^,*j*] are now forbidden and we remove them from $\mathcal {S}$. We repeat the steps (i) and (ii) until there is no remaining couple in the set $\mathcal {S}$ with BLAST bit-score similarity at least *ℓ*. Note that, in the process of seed generation, when there are several couples with the same sequence similarity, we randomly pick one of them.

Algorithm 2 describes the SeedGeneration algorithm in detail. In this algorithm, for a set of couples $\mathcal {A}$, $V_{1}(\mathcal {A})$ defines the set of nodes from network *G*_1_ in $\mathcal {A}$, i.e., $V_{1}(\mathcal {A}) = \lbrace i \vert \exists j \ {\mathrm {s.t.}} \ [\!i,j]\in \mathcal {A}~\text {for some} j\rbrace $. We define $V_{2}(\mathcal {A})$ similarly. Also, *B* *l* *a* *s* *t* *B* *i* *t*(*i*,*j*) denotes the BLAST bit-score similarity between two proteins *i* and *j*.

A priori, the probability of biological similarity of a protein couple decreases with a decrease in the sequence similarity. Therefore, there is a trade-off between the number of protein couples with the same biological functions and the accuracy (i.e., the ratio of couples with the same functions over the size of seed set) based on *ℓ*. Clearly, choosing a high value for *ℓ* aligns proteins that, with a high probability, have similar functions. However, this can result in removing couples with lower sequence similarities, but the same functions from the initial seed-set.



The second step of PROPER (the MapPercolation algorithm) starts the alignment process from the seed couples (set $\mathcal {A}$) obtained from the set of pairwise similarities $\mathcal {S}$ (see the SeedGeneration algorithm). It then incrementally generates the set *π* of matched couples among $V_{1} \times V_{2} \setminus \mathcal {A}$. In the MapPercolation step, the PROPER algorithm relies only on the structure of *G*_1,2_, and it does not use the sequence similarities. In this regard, the seed couples are added to the set of aligned couples *π*. Then, at each time-step, the goal of the PGM algorithm is to add a new couple to the set *π* so that structural similarity is maximized.

In the process of the MapPercolation algorithm, we look at the neighboring couples of the previously matched couples. We say a couple of proteins [ *i* ^′^,*j* ^′^]∈*V*_1_×*V*_2_ is a neighbor of another couple [ *i*,*j*] if and only if (*i*,*i* ^′^)∈*E*_1_ and (*j*,*j* ^′^)∈*E*_2_. Indeed, the evidence for deciding which couple to match (called the score of a couple) is the number of common neighbors each couple has in the set of currently aligned couples. To achieve the maximum structural similarity, our algorithm chooses the next couple in a greedy way: it chooses the couple with the maximum number of common neighbors *s* *c* *o* *r* *e*_*max*_ (provided there are at least *r*) in *π* and permanently aligns them. Therefore the number of conserved interactions by adding the couple with the highest score is *s* *c* *o* *r* *e*_*max*_. Note that a new couple of proteins can be matched if its score is at least *r*.

**Fig. 1:**
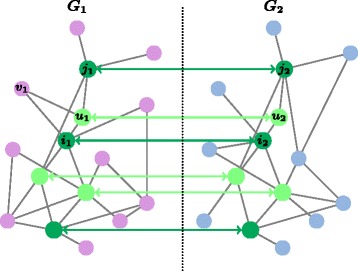
*Dark-green nodes* correspond the initial seed-set. Couples [ *i*_1_,*i*_2_],[ *i*_1_,*j*_2_],[*j*_1_,*j*_2_],[*j*_1_,*i*_2_],[ *v*_1_,*i*_2_],[ *v*_1_,*j*_2_] are neighboring couples of the couple [ *u*_1_,*u*_2_]. The couples [*i*_1_,*i*_2_] and [*j*_1_,*j*_2_] are the common neighbors of the couple [ *u*_1_,*u*_1_] in the set of already matched couples *π*, i.e., the score of couple [ *u*_1_,*u*_2_] is two. *Light-green nodes* are the nodes that are matched after the first three steps of the MapPercolation algorithm



When there are several couples with the maximum score, we tie-break by the minimum degree-difference in the two networks, i.e., we choose the couple [ *i*,*j*] with the minimum |*d*_1,*i*_−*d*_2,*j*_|, where *d*_1,*i*_ and *d*_2,*j*_ denote the degrees of nodes *i* and *j* in the networks *G*_1_ and *G*_2_, respectively. The proteins with closer number of interactions (i.e., closer degrees) have more structural similarity. If there is more than one couple with the minimum degree difference, we choose the couple with the minimum *d*_1,*i*_+*d*_2,*j*_. The couple with the minimum *d*_1,*i*_+*d*_2,*j*_ minimizes the number of mismatched interactions. Finally, if there are still several candidate couples, we randomly pick one of them. The process of alignment continues to the point where there is no remaining unmatched couple of proteins (we say a couple [ *i*,*j*] is unmatched if *i*∉*V*_1_(*π*) and *j*∉*V*_2_(*π*)) with at least *r* common neighbors, in the current set of aligned proteins. Note that for a given value of *r*, only nodes with degree at least *r* can get enough score to be matched. More precisely, MapPercolation is not able to align: (i) unmatched nodes with a degree less than *r*, and (ii) couples that have not gained enough scores. Figure 1 presents an example of the second step of PROPER (the MapPercolation algorithm). Algorithm 2 describes this algorithm.

### Performance measures

In this section, we explain the measures used for comparing alignment algorithms. As there is no single standard measure for evaluating the quality of alignments, we use several existing measures [20, 21, 46]. In addition, we introduce a new measure for comparison based on the performance of algorithms in aligning biological pathways.

For better illustration, in this section we assume that, without loss of generality, *G*_2_ has at least as many nodes as *G*_1_, i.e., |*V*_1_|≤|*V*_2_|. Let *π* denote the mapping produced by an alignment algorithm. Also, let *G*[ *U*] denote the induced subgraph of *G* on a set of vertices *U*. Assume *π* maps the nodes $V_{1}^{\prime } \subset V_{1}$ to the nodes $V_{2}^{\prime } \subset V_{2}$, i.e., $V_{2}^{\prime } = \pi \left (V_{1}^{\prime }\right)$. Note that many global alignment algorithms do not match all the nodes from graph *G*_1_ to a node from graph *G*_2_, i.e., they align a large fraction of the nodes but not all of them. We define graph *G*_0_(*V*_0_,*E*_0_) as the intersection of the two graphs *G*_1_ and *G*_2_ under the alignment *π*, i.e., *V*_0_ is the set of proteins in graph *G*_1_ aligned by *π* to a protein in graph *G*_2_; and *E*_0_ is the set of interactions in *G*_1_, conserved under the alignment *π* in the graph *G*_2_. Formally, we have $V_{0} = V_{1}^{\prime }$ and $E_{0} = E_{G_{1}[\!V_{1}^{\prime }]} \cap \pi ^{-1}(E_{G_{2}[\!V_{2}^{\prime }]}$).

#### Structural and functional similarity measures

In this section, we review the measures that are used widely to evaluate the performance of network alignment algorithms.

(i) Node correctness (NC) of an alignment is defined as the ratio of the number of correctly aligned couples to the number of nodes in the smaller network (i.e., |*V*_1_|) [30]. The precision is defined as the ratio of number of correctly aligned couples to the total number of couples |*π*| in the alignment *π*. These measures are applicable only to synthetic networks, because they can be used only for alignments with known ground-truth [21].

As the true alignment between the proteins of two species is not known completely for real networks, it is not possible to directly calculate the NC and precision of an alignment [20, 21, 46]. To compare the performance of algorithms over real datasets, two different types of measures were introduced in the literature. The first group of measures uses the topological similarity of aligned networks. The second group measures the quality of an alignment by using other biological information.

The following measures are used for evaluating the topological similarity of aligned networks.

(ii) The number of conserved interactions under the alignment *π* (call it *Δ*_*π*_) is one of the measures used to evaluate the quality of algorithms based on the topological similarity [1]. Formally,

$$\begin{array}{*{20}l} \Delta_{\pi} = |\pi(E_{1}) \cap E_{2} |. \end{array} $$

(iii) Edge correctness (EC) is a measure of topological similarity among the aligned networks [30]. EC computes the ratio of edges from graph *G*_1_, i.e., all the edges in the smaller network, which are conserved under the alignment *π*. Formally,

$$\begin{array}{*{20}l} \text{EC} = \frac{|\pi(E_{1}) \cap E_{2} |}{|E_{1}|}. \end{array} $$

(iv) Recall that the numbers of proteins (nodes) in the two networks are not equal. Therefore, one drawback of the EC measure is that aligning sparse regions of *G*_1_ with dense regions of *G*_2_ can result in high values of EC. The induced conserved-structure score (*ICS*) measures the structural similarity of aligned networks by penalizing dense regions of *G*_2_ [41]. The ICS score for an alignment *π* from graph *G*_1_ with graph *G*_2_ is

$$\begin{array}{*{20}l} ICS = \frac{|\pi(E_{1}) \cap E_{2}|}{|E_{G_{2}\left[\pi(V_{1})\right]}|}. \end{array} $$

(v) The symmetric substructure score (*S* ^3^) is defined with respect to both *G*_1,2_ networks [33]. The *S* ^3^ measure penalizes the alignments that map sparse regions of one network to denser regions of the other network. Formally, *S* ^3^ is defined as follows.

$$\begin{array}{*{20}l} S^{3} = \frac{|\pi(E_{1}) \cap E_{2}|}{|E_{1}| + |E_{G_{2}\left[\pi(V_{1})\right]}| - |\pi(E_{1}) \cap E_{2}|}. \end{array} $$

Note that |*E*_1_| refers to all the edges in the smaller network.

(vi) The largest connected shared-component (LCSC) is the largest connected subgraph of *G*_1_, which is found to also exist in *G*_2_, i.e., the largest connected component in graph *G*_0_ [46]. Let |*L* *C* *S* *C*| denote the number of nodes in LCSC. Also, the share of nodes in LCSC is defined as $\frac {|LCSC|}{|V_{1}|}$ [16].

We now introduce the second group of measures that are used for evaluating the biological quality of alignments.

(vii) The gene-ontology consistency (GOC) score measures the functional similarity of aligned proteins. Note that usually more than one gene ontology (GO) terms are assigned to a protein [58]. Also, as the GO datasets are noisy and proteins have diverse functions, it is possible that true ortholog proteins do not have exactly the same set of GO terms. GOC for an aligned couple of proteins *u*∈*V*_1_ and *v*∈*V*_2_ is defined as the Jaccard similarity coefficient between the GO terms of the two proteins [35]. Formally, it is defined as

$$\begin{array}{*{20}l} GOC(u,v) = \frac{|GO(u) \cap GO(v)|}{|GO(u) \cup GO(v)|}, \end{array} $$

where *G* *O*(*u*) denotes the set of GO terms associated with the protein *u*. *G* *O* *C*(*π*) is calculated by summation over the GOC terms of all the aligned couples in *π*:

1 $$\begin{array}{*{20}l}  GOC(\pi) = \sum_{u \in V_{1}} GOC(u,\pi(u)). \end{array} $$

For ease of notation we refer to *G* *O* *C*(*π*) as GOC score.

(viii) To compare algorithms based on the sequence similarities of aligned proteins, we use a slightly modified version of the average normalized bit–score (ANBS) measure proposed in [59]. ANBS for two graphs *G*_1_(*V*_1_,*E*_1_) and *G*_2_(*V*_2_,*E*_2_) under the alignment *π* is defined as follows.

$$\begin{array}{*{20}l} & ANBS(\pi) = \\ & |V_{1}|^{-1} \sum_{i \in V_{1}{(\pi)}} \frac{BlastBit(i,\pi(i))}{\sqrt{BlastBit(i,i)BlastBit(\pi(i),\pi(i))}}. \end{array} $$

#### Pathway comparison measures

In order to evaluate the performance of algorithms in aligning biological pathways, we introduce a new measure in this section. This measure captures the quality of alignments based on a higher level of functional and structural similarities (beyond the introduced measures such as the similarity of GO terms and the number of conserved interactions).

It is known that there are many biological pathways with similar functions in different species [12]. The KEGG PATHWAY database [60] provides a set of experimentally found biological pathways. In this database, a pathway is called by the name of a species (e.g., hsa for Homo sapiens), followed by a number. The pathways with the same number have the same function in different species. For example, hsa03040, mmu03040, dme03040 and sce03040 are in Homo sapiens (human), Mus musculus (mouse), Drosophila melanogaster (fruit fly) and Saccharomyces cerevisiae (budding yeast), respectively. These pathways have the same functions.^2^ Assume *P* *W*_*i*,1_ denotes the set of proteins from a pathway with number *i* in the PPI network of the first species (i.e., *G*_1_). Similarly, we define *P* *W*_*i*,2_. For pathway *i*, *Δ*_*π*,*i*_ denotes the number of conserved interactions between the proteins in this pathway under the alignment *π*, i.e., $\Delta _{\pi,i} = E_{G_{1}[PW_{i,1}]} \cap \pi ^{-1}(E_{G_{2}[PW_{i,2}]})$. Note that we are looking for pathways that are present in both aligned species.

We say a protein *u* from a pathway is aligned correctly, if it is mapped to a protein *v* from a pathway with the same function. For pathway *i*, we define the number of correctly mapped proteins as |*P* *W*_*i*,1_∩*π* ^−1^(*P* *W*_*i*,2_)|. This measure corresponds to the number of proteins that, from pathway *i* in the first species, are mapped to a protein from the same pathway in the second species. For pathway *i*, we define the accuracy as

2 $$\begin{array}{*{20}l}  acc_{\pi, i} = \frac{2 |PW_{i,1} \cap \pi^{-1}(PW_{i,2})|}{|PW_{i,1}| + |PW_{i,2}|}. \end{array} $$

This measure corresponds to the fraction of correctly mapped proteins in pathway *i*.

We conjecture that a good alignment algorithm should align proteins from pathways with the same functions across species, and many interactions among these proteins are conserved. To quantify this expectation, we set a threshold over the structural similarity of aligned pathways to consider them as a correct alignment. We say that an alignment *π* successfully aligns a pathway *i*, if there are at least *δ* conserved interactions under the alignment *π* for proteins in that pathway, i.e., if *Δ*_*π*,*i*_≥*δ*. This thresholding guarantees that the structural similarity of aligned pathways are more than a minimum value (here, *δ* conserved interactions). To evaluate the performance of an algorithm based on this thresholding criterion, we define a set of measures as follows.

1. We consider pathways with at least *δ* (say *δ*≥2) interactions in each of the species. Let “ *#*PW_*δ*_” denote the number of such pathways.
2. Alignment *π* successfully aligns pathway *i*, if *Δ*_*π*,*i*_≥*δ*. The variable “ *#*FPW_*δ*_” refers to the number of successfully aligned pathways. We define the recall as
  3 $$\begin{array}{*{20}l}  recall_{\pi, \delta} = \frac{\#\text{FPW}_{\delta}}{\# \text{PW}_{\delta}}. \end{array} $$
3. Again, for a correctly aligned pathway i, we define *a* *c* *c*_*π*,*δ*,*i*_ similar to (2).

**Fig. 2:**
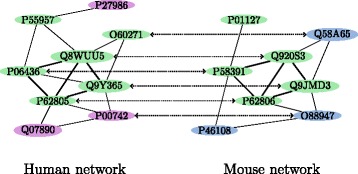
In this figure, two example PPI networks are given. *Green nodes* are proteins which are in the same pathway (i.e., a pathway with the same number in both species). *Dotted lines* represent the alignment *π* between these two networks. Under this alignment, there are five conserved interactions between proteins in this pathway (shown by *thick black edges* in each network). Also, the number of correctly mapped proteins is four. Therefore, the accuracy of aligning this pathway is $acc_{\pi,i} = \frac {2 \times 4}{6 + 5}$, where there are six and five proteins from this pathway in each species, respectively

The averages over all *i* of all the *a* *c* *c*_*π*,*i*_ and *a* *c* *c*_*π*,*δ*,*i*_ values are represented by $\overline {acc_{\pi }}$ and $\overline {acc_{\pi, \delta }}$, respectively. Figure 2 provides a toy example of how to calculate the pathway alignment measures.

## Results

In this section, we compare PROPER with the main state-of-the-art network alignment algorithms, specifically (i) with L-GRAAL as the most recent member of GRAAL family that takes into account both sequence and structural similarities [23]; (ii) with MAGNA++ that tries to maximize one of the EC, ICS or *S* ^3^ measures [33, 34] (In our experiments we run MAGNA++ in two different modes of maximizing *S* ^3^, which is the superior mode for MAGNA++ [33], and EC); (iii) with IsoRank [28] as one of the first global PPI-network alignment algorithms; (iv) with PINALOG [36]; and (v) with SPINAL I and II [35] as their performances are reported to be among the best alignment-algorithms [46]. Table 1 provides an overview of the arguments and parameters of the algorithms used in our comparisons. Note that it is recommended to use SPINAL and MAGNA++ in modes I and *S* ^3^, respectively. Also, the recommended settings for IsoRank is *α*=0.6. For the other algorithms, no default setting is provided. We evaluate the performance of PROPER with *r*=1 and different values of *ℓ*.

**Table 1:** Algorithms and their parameters

Algorithm	Commandline arguments	Parameters
IsoRank [28]	–K 50 –thresh 1e-5 –alpha *α* –maxveclen 1000000	*α*∈{0.3,0.5,0.6,0.7}
PINALOG [36, 77]	do not require arguments	none
L-GRAAL [23]	–a *α* –I 50	*α*∈{0.3,0.5,0.7}
MAGNA++(*S* ^3^) [34]	–m S3 –p 1000 –n 15000 –f 5 –a *α* –t 16	*α*∈{0.3,0.5,0.7}
MAGNA++(EC) [34]	–m EC –p 1000 –n 15000 –f 5 –a *α* –t 16	*α*∈{0.3,0.5,0.7}
SPINAL I [35]	–mode -I –alpha *α*	*α*∈{0.3,0.5,0.7}
SPINAL II [35]	–mode -II –alpha *α*	*α*∈{0.3,0.5,0.7}

All the algorithms use two sets of data as input: (i) the PPI networks of two species, and (ii) the pairwise BLAST similarities (in form of BLAST bit-score) between proteins from the first species and proteins from the second species. We use two different PPI-network databases for our comparisons. The first one is from IntAct molecular interaction database [9, 61]. This database enables us to compare algorithms based on large and more recent PPI networks. The GO annotation terms are extracted from the Gene Ontology Annotation (UniProt-GOA) Database [62, 63]. For pathway comparisons over these networks we can use data from [60]. The second database is Isobase [64], a common dataset used in comparison of recent algorithms [20, 46]. The results for experiments over Isobase dataset are provided in Additional file 1. For further evaluations, we use synthetic networks with a known ground-truth.

### Structural and functional based comparisons

Table 2 provides a brief description of the PPI networks for five major eukaryotic species, namely C. elegans (ce), D. melanogaster (dm), H. sapiens (hs), M. musculus (mm) and S. cerevisiae (sc); they are extracted from the IntAct database [9, 61]: The last column of Table 2 shows the number of pathways of each species from KEGG PATHWAY database [60]. The amino-acid sequences of proteins for each species are extracted in the FASTA format from UniProt database [65, 66]. The BLAST bit-score similarities [47] are calculated using these amino acid sequences.

**Table 2:** PPI networks of five major eukaryotic species from IntAct molecular interaction database [9, 61]

Species	*#*nodes	*#*edges	Avg. deg.	*#*pathways
C. elegans	4950	11550	4.67	117
D. melanogaster	8532	26289	6.16	127
H. sapiens	19141	83312	8.71	288
M. musculus	10765	22345	4.15	284
S. cerevisiae	6283	76497	24.35	98

**Fig. 3:**
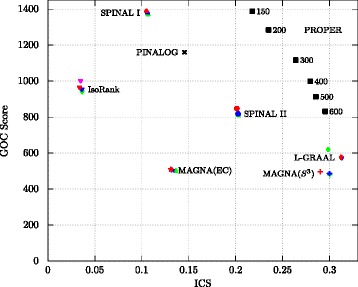
Comparison of different global network aligners based on the average GO consistency vs. average integrated conserved structure score. For the PROPER algorithm, we set *r*=1 and each point corresponds to a different value of *ℓ*. Also, the *red, blue, magenta and green points* correspond to the parameters *α*=0.3,0.5,0.6 and 0.7, respectively

Figure 3 compares algorithms based on the average ICS versus average GOC score for all the possible 10 pairwise alignments between the species. We observe that PROPER outperforms the other algorithms in both measures, i.e., the PROPER algorithm finds alignments with higher functional (GOC score) and structural (ICS) similarities. For the detailed comparisons of the algorithms refer to Figs. 6, 7, 8 and Additional files 1 and 2.

**Fig. 4:**
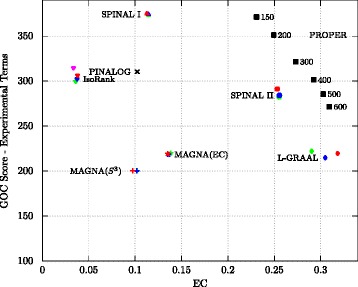
Comparison of different global network aligners based on the average GO consistency (by considering only experimentally verified GO terms) vs. EC score. For the PROPER algorithm, we set *r*=1 and each point corresponds to a different value of *ℓ*. Also, the *red, blue, magenta and green points* correspond to the parameters *α*=0.3,0.5,0.6 and 0.7, respectively

Note that many of the GO annotations are based on only sequence similarities, and these annotations could increase the GOC scores artificially. Clark and Kalita [46] (similar to [35]) propose to also compare algorithms by using only the experimentally verified GO terms (along the comparisons based on all the GO terms) to eliminate the effects of sequence similarities in the GOC evaluations. For this reason, in our next experiment, we consider only GO terms with codes “EXP”, “IDA”, “IMP”, “IGI”, “IEP” and “IPI” (the codes for experimental GO terms), and we exclude the annotations derived from computational methods. Figure 4 compares the GOC (based on experimentally verified GO terms) versus EC score. The result of this experiment confirms the superiority of PROPER over the other algorithms.

**Fig. 5:**
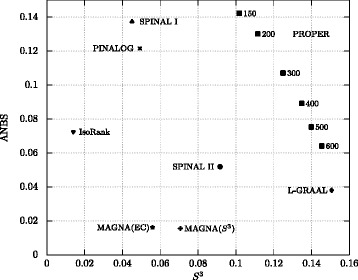
Comparison of different global network aligners based on the average ANBS vs. average *S* ^3^ score. For the PROPER algorithm, we set *r*=1 and each point corresponds to a different value of *ℓ*. The parameter *α* is 0.7

Figure 5 evaluates the performance of algorithms based on *S* ^3^ (for structural similarity) and ANBS (for functional similarity) measures. Again, the PROPER algorithm performs the best based on the two measures, simultaneously.

Table 3 reports the average number of aligned couples and the average of share of nodes in LCSC. We observe that MAGNA++ and IsoRank find, irrespective of the similarity of networks, alignments with full coverages, i.e., the size of their alignments is equal to the number of nodes in the smaller network; and PINALOG has the lowest coverage among the algorithms. The size of an alignment alone is not a good indicator of its quality, because an algorithm with a large coverage might find alignments with low functional-similarities and structural-similarities. Instead, we can consider the sum of functional similarities of aligned proteins. To address this point, for example, GOC score (1) captures the total functional similarity, by summation over all the couples in *π* (see Figs. 3 and 4). We can also consider the size of shared structure between networks. To address this second point, we use LCSC. A larger LCSC implies that we have found a larger amount of shared structure between the two PPI networks [16]. From Table 3, we observe that PROPER, L-GRAAL and SPINAL II outperform the other algorithms (with huge margins), based on the share of nodes in LCSC.

**Table 3:** The average number of aligned couples (i.e., |*π*|) and the average of share of nodes in LCSC (i.e., |*L* *C* *S* *C*|/|*V*_1_|). We use *α*=0.7 for SPINAL, IsoRank, MAGNA and L-GRAAL, and *r*=1 for PROPER

Algorithms	|*π*|	|*L* *C* *S* *C*|/|*V*_1_|
PROPER (*ℓ*=150)	5521.2	0.528
PROPER (*ℓ*=600)	5320.5	**0.728**
SPINAL I	6364.3	0.219
SPINAL II	6433.4	0.720
PINALOG	3740.9	0.233
L-GRAAL	5616.4	0.726
MAGNA++(*S* ^3^)	**6647.8**	0.292
MAGNA++(EC)	**6647.8**	0.353
IsoRank	**6647.8**	0.051

The best value for each column is highlighted in boldface

**Fig. 6:**
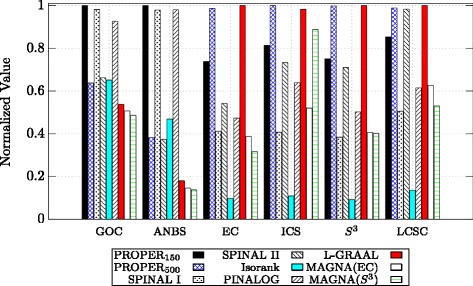
Comparison of different global network aligners on aligning H. sapiens and S. cerevisiae based on six different measures. For the PROPER algorithm, we set *r*=1 and *ℓ*∈{150,500}. The parameter *α* is 0.7

**Fig. 7:**
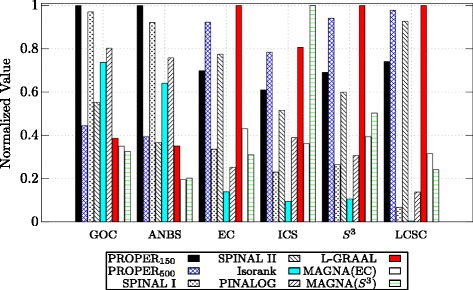
Comparison of different global network aligners on aligning C. elegans and D. melanogaster based on six different measures. For the PROPER algorithm, we set *r*=1 and *ℓ*∈{150,500}. The parameter *α* is 0.7

**Fig. 8:**
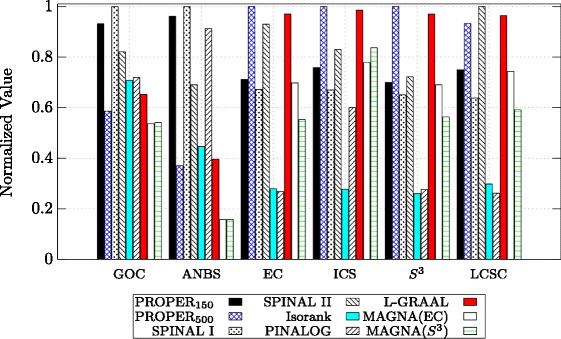
Comparison of different global network aligners on aligning M. musculus and S. cerevisiae based on six different measures. For the PROPER algorithm, we set *r*=1 and *ℓ*∈{150,500}. The parameter *α* is 0.7

Figure 6 provides a detailed comparison between the algorithms based on their performance in aligning H. sapiens with S. cerevisiae. Also, detailed comparisons between C. elegans and D. melanogaster, and M. musculus and S. cerevisiae are provided in Figs. 7 and 8, respectively. Note that in Figs. 6, 7 and 8, the values for each measure are normalized to the highest value, i.e., for each measure, in these figures, the maximum is 1 for the best algorithm and values for the other algorithms are normalized with respect to the maximum. We observe that PROPER outperforms the other algorithms in terms of most of GOC, ANBS, ICS, *S* ^3^, EC and LCSC measures.

### The MapPercolation algorithm and ***r***

The PROPER algorithm has two main steps: (i) SeedGeneration and (ii) MapPercolation. The numbers of aligned couples in the first and second steps depend on *ℓ* and *r*, respectively. In Table 4, we report the average number of aligned couples (i.e., |*π*|) in the first and second steps of PROPER for different values of *ℓ* and *r*∈{1,2}. We observe that by increasing the value of *ℓ*, the number of aligned couples in the first step decreases. This is because the number of couples with BLAST bit-score of at least *ℓ* has an inverse relationship with *ℓ*. In the second step, |*π*| increases by a factor of 2.5 to 7.6 for *ℓ*∈{150,200,300,400,500,600} with *r*=1. For the detailed experimental result of PROPER with *r*∈{1,2} refer to Table S1 in Additional file 2.

**Table 4:** The average number of aligned couples when running (i) only the first step of PROPER (i.e., the SeedGeneration algorithm), and (ii,iii) PROPER with *r*={1,2} with different values of *ℓ*

*ℓ*	SeedGeneration	*r*=2	*r*=1
150	2198.4	3116.1	5521.2
200	1875.6	2900.3	5471.4
300	1393.9	2618.4	5432.9
400	1083.1	2408.7	5416.4
500	861.0	2216.1	5347.4
600	696.4	2094.0	5320.5

**Fig. 9:**
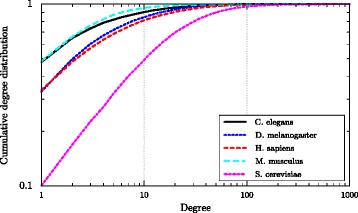
Cumulative degree distribution for all the networks from Table 2

Choosing smaller values of *r* reduces the required structural similarity for aligning a couple. This explains why the number of aligned couples for *r*=1 is larger than for *r*=2 in Table 4. Note that the MapPercolation algorithm, for a given value of *r*, cannot align nodes with degrees less than *r*. From Fig. 9, which reports the degree distribution of different networks, we observe that there are many nodes with degree one, e.g, almost half of nodes for C. elegans and M. musculus. These nodes of degree one can not be aligned with *r*=2, and this is the reason we choose *r*=1 for our experiments. In general, the value of *r* controls the strength of the structural evidence required before we decide to align a couple and a larger *r* makes errors less likely. We believe that by the increasing number of known PPI interactions over time, which consequently results in a decrease of the number of low-degree nodes, a larger value of *r* will generate better alignments.

### Synthetic networks

In this section, we compare algorithms based on their performance over synthetic networks. For this, we consider the high-confidence yeast Saccharomyces cerevisiae PPI network with 1004 nodes and 8323 edges [33, 67]; this network serves as our “ground-truth”. For this experiment, a noisy version of the yeast network is generated by sampling each of its nodes and interactions with a probability *s*. Here, *s* controls the similarity of a sampled network with the original network, and we take 1−*s* as the “level of noise”. Also, the sequence similarity for a subset of randomly chosen proteins is provided as a side information. In this experiment, the ground-truth node mapping is known by design, which enables us to calculate NC and precision. Note that in order to account for the randomness of our experiments, we provide the average of 50 different alignments for each level of noise and available sequence similarity.

**Fig. 10:**
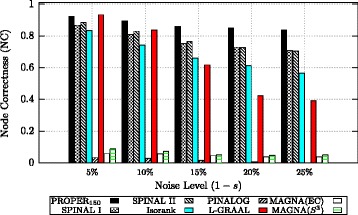
Comparison of different global network aligners over synthetic networks based on node correctness (NC). The sequence similarity for 50% of randomly chosen proteins is provided. For the PROPER algorithm, we set *r*=1 and *ℓ*=150. The parameter *α* is 0.7

In the first experiment, we align the original network with five networks that are generated by different levels of noise 1−*s*∈{5*%*,10*%*,15*%*,20*%*,25*%*}. Also, the sequence similarity for 50% of randomly chosen proteins is provided. Figure 10 provides NC comparison over these synthetic networks for different levels of noise. From Fig. 10, for example, we observe that PROPER aligns networks which are sampled with the noise level 1−*s*=15*%* with NC=0.86. Note that the average number of nodes for different noise levels (from 5 to 25%) is 946.48, 893.24, 832.54, 780.4 and 730.96, respectively. This means that PROPER correctly aligns 0.86×832.54≈716 couples. Figure S1 in Additional file 2 compares algorithms based on precision. From the result of this experiment, we observe that for a low level of noise (1−*s*=5*%*) L-GRAAL has the best performance and PROPER comes second. With increasing level of noise, the performance of PROPER remains almost unaffected, whereas the quality of the other alignments decreases quite markedly.

**Fig. 11:**
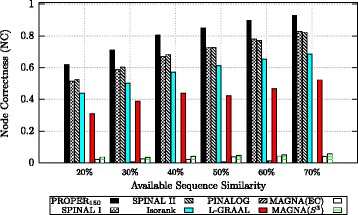
Comparison of different global network aligners over synthetic networks based on node correctness (NC). The level of noise is set to 1−*s*=20*%*. For the PROPER algorithm, we set *r*=1 and *ℓ*=150. The parameter *α* is 0.7

In the second experiment, we investigate the effect of available sequence similarity on the performance of algorithms. We consider different amounts of available sequence similarity and fix the level of noise to 1−*s*=20*%*. Figure 11 compares algorithms when the sequence similarities for 20*%*,30*%*,40*%*,50*%*,60*%* and 70% of randomly chosen proteins are provided. Figure S2 in Additional file 2 compares algorithms based on precision. We observe that PROPER outperforms the other algorithms over the entire range of available sequence similarities.

These two experiments confirm the success of the PROPER algorithm in aligning synthetic networks and its robustness to high levels of noise.

### Aligning biological pathways

In this section, we compare algorithms based on their performance in aligning biological pathways. We use *α*=0.7 for SPINAL, IsoRank, MAGNA and L-GRAAL, and *r*=1,*l*=150 for PROPER. We use the measures introduced in the Pathway comparison measures section. For our comparisons, we consider the alignment of H. sapiens with the other four species from Table 2. We know that there are several proteins that belong to more than one pathway, because some proteins could be involved in different biological processes. For this reason, along the results for all the pathways, we consider a subset of non-overlapping pathways for each pair of species. Table 5 reports the number of common KEGG pathways between different pairs of species, where we consider (i) all the pathways, (ii) pathways with at least *δ*=4 interactions in each of the species, and (iii) a subset of non-overlapping pathways.

**Table 5:** Number of common KEGG pathways between different pairs of species

Pair of species	#PW	#PW(*δ*=4)	#PW (no-overlap)
hs-ce	116	19	37
hs-dm	122	31	40
hs-mm	283	152	49
hs-sc	98	32	34

For the first experiment, we do not consider the topological similarities of aligned pathways. The result for alignments of pathways from different algorithms is provided in Table 6. We observe that PROPER outperforms the other algorithms in terms of accuracy. In the second experiment, for each algorithm we consider only the pathways with at least *δ*=4 conserved interactions across species (i.e., *Δ*_*π*,*i*_≥4). Table 7 provides the results for this case. Again, we observe that the PROPER algorithm outperforms the other algorithms, i.e., on average it aligns more pathways with a higher accuracy. MAGNA++ performs very poorly in this experiment and we omit it from Table 7.

**Table 6:** Comparison of algorithms based on aligning biological pathways. This table reports the average value of $\overline {acc_{\pi }}$ for pairwise alignments between Home sapiens and the four other species from Table 2

Algorithms	$\overline {acc_{\pi }}$	$\overline {acc_{\pi }}$ (no-overlap)
PROPER	**0.471**	**0.442**
SPINAL I	0.447	0.426
SPINAL II	0.115	0.134
PINALOG	0.409	0.397
L-GRAAL	0.232	0.218
MAGNA++(*S* ^3^)	0.016	0.020
MAGNA++(EC)	0.017	0.020
IsoRank	0.202	0.195

The best value for each column is highlighted in boldface

**Table 7:** Comparison of algorithms based on pathway alignment measures for *δ*=4 (i.e., *Δ*_*π*,*i*_≥4). This table reports the average value of measures for pairwise alignments between Home sapiens and the four other species from Table 2

Algorithms	*#*FPW	$\overline {acc_{\pi, \delta }}$	*r* *e* *c* *a* *l* *l*_*π*_
PROPER	**42.5**	**0.585**	**0.584**
SPINAL I	38.75	0.554	0.536
SPINAL II	9.0	0.223	0.102
PINALOG	39.75	0.497	0.547
L-GRAAL	25.5	0.320	0.235
IsoRank	18.5	0.356	0.225

The best value for each column is highlighted in boldface

**Fig. 12:**
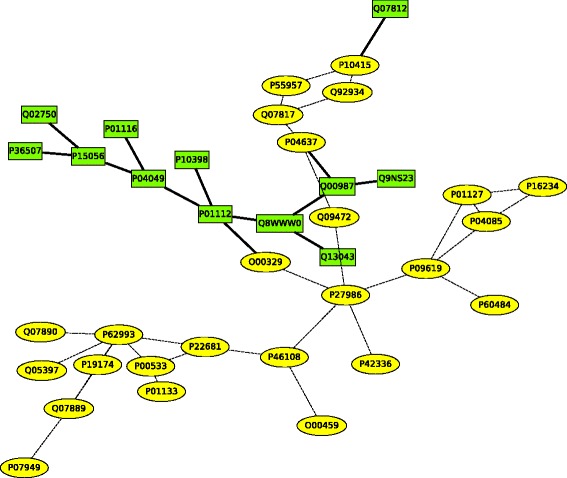
The connected subgraph of hsa05200 and mmu05200 pathways in human and mouse from the PROPER algorithm, with conserved interactions in both species. This connected subgraph has 37 nodes and 42 edges. The PINALOG algorithm returns the second largest connected subgraph. The rectangular nodes and solid edges are the proteins and interactions among them that are found only by the PROPER algorithm

For many pathways, the PROPER algorithm, compared to other algorithms, returns alignments with a larger portion of connected conserved subgraphs. For example, Fig. 12 shows the connected conserved subgraph of pathways hsa05200 and mmu05200 between human and mouse^3^ The connected subgraph of this pathway has 37 nodes and 42 edges, which is larger than alignments by the other algorithms (see Additional file 3 for the detailed comparison results).

### Execution time

A fast and scalable alignment algorithm is needed with the growing size of PPI networks. One of the key features of the PROPER algorithm is its low computational complexity and scalability. PROPER is able to align synthesis networks with millions of nodes in less than a hour. In fact, the complexity of our algorithm is *O*((|*E*_1_|+|*E*_2_|) min(*D*_1_,*D*_2_)), where *D*_1,2_ are the maximum degrees in the two networks. Table 8 provides the total execution time of algorithms for 10 pairwise alignments between the five species from Table 2. All computations are done on the same Linux machine with 16 GB of memory and 8 Intel Xeon E3-1270 CPUs working at clock speeds 3.50 GHz. We observe that PROPER runs much faster than the other algorithms.

**Table 8:** The total execution time of algorithms for 10 pairwise alignments between the five species from Table 2

Aligner	Time
PROPER	317 s
L-GRAAL	4 h and 2 min
MAGNA++(*S* ^3^)	7 h and 47 min
MAGNA++(EC)	7 h and 41 min
PINALOG	2 days, 5 h and 26 min
SPINAL I	10 h and 51 min
SPINAL II	11 h and 56 min
IsoRank	12 h and 43 min

## Discussion

The purpose of network alignment algorithms is to find functional and structural similarities between PPI networks of different species [21]. Most of the works in the literature model global network alignment as an optimization problem over the convex combination of sequence and structural similarities between two networks [1, 28, 30]. This class of algorithms aims to maximize a cost function in order to increase the following two quantities simultaneously: (i) the pairwise similarities between aligned proteins (e.g., by maximizing the summation over all the BLAST similarities of aligned proteins), and (ii) the structural similarity between the two graphs, (e.g., by maximizing the conserved PPIs under the alignment) [46].

It appears that this particular formulation of the optimization problem precludes these algorithms from making good alignments by using both similarities jointly [46]. For example, the authors of [40] have shown that in the IsoRank algorithm for the structure-only (*α*=1) alignment, the similarity of two nodes is only a function of their degrees. Their results explicate the poor performance of IsoRank in finding alignments with good structural similarities. Also, our experimental results confirm the trade-off between structural and functional similarities in most of the state-of-the-art network alignment algorithms. We observe that each of the five algorithms evaluated here, namely L-GRAAL, MAGNA++, IsoRank, PINALOG and SPINAL, covers only a small portion of the trade-off frontier (see Figs. 3 and 4). In summary, we believe that these observations make it necessary to study the PPI network alignment problem under rigorous mathematical models.

The PROPER algorithm, in comparison, shows less compromise between the functional similarities among aligned proteins and the topological similarity. Figures 3, 4 and 5 show that our algorithm sweeps the frontier (i.e., has the best trade-off between both measures) more robustly than the other algorithms. In addition, large conserved subgraphs with the same function are aligned with PROPER. The PROPER algorithm not only aligns proteins and their corresponding interactions from two different species better than other algorithms, it also aligns the conserved pathways between the species with higher accuracy. This shows that instead of finding conserved single pairwise PPIs, PROPER represents a more biologically realistic performance by detecting sub-networks of conserved interactions from pathways with the same function among species.

In addition to its superior accuracy, PROPER performs better in terms of memory usage and speed, because the alignment process of PROPER is a very simple local propagation method.

### Why the PROPER algorithm?

In the following, we provide two reasons why PROPER performs well in terms of the cost functions considered.

The first reason is that a high BLAST bit-score is a reliable indicator of a match, whereas a low BLAST bit-score is very unreliable for many functional characteristics [68]. As a consequence, rather than optimizing a convex combination of functional similarity with structural similarity, it is advantageous to ascribe high confidence to the sparse set of high-BLAST couples, and to completely ignore low BLAST bit-scores. This is what PROPER does, by generating an initial seed-set of high BLAST couples, and then by propagating outwards from this seed set as a purely structure-driven process. Note that as the PGM class of algorithms are shown to be robust against noise in the seed set [45], PROPER is not too sensitive to the sequence similarity threshold *ℓ* for aligning new couples of proteins.

The second reason is more speculative and has to do with the statistical structure of the two networks being matched. Computational biology postulates evolutionary models to explain the difference between PPI networks. Studies have identified gene duplication and the gain or loss of genes and their interactions as the key evolutionary events in forming biological networks [69–71]. Several evolutionary models for regulatory networks and protein–interaction networks have been introduced based on these observed evolutionary processes [72–74].

Percolation-based methods for network alignment are well-suited for network pairs whose structural differences arise from the random deletions of nodes and edges. Specifically, in prior works, the authors of [42, 43] define the *G*(*n*,*p*;*t*,*s*) random bigraph model for generating two correlated networks *G*_1,2_ that rely on node and edge sampling processes. The two parameters *t* and *s* control the node and edge similarity of the generated graphs. Although the analysis in these prior works is for a different algorithm within the PGM class, we believe the main concepts carry over to PROPER.

More specifically, for the sake of simplicity, we assume that the evolutionary process can only delete proteins and interactions among proteins. We call this model *E* *v* *o* *l* *v* *e*(*G*,*t*,*s*), where we postulate an ancestor network *G*(*V*,*E*), from which both observable networks *G*_1,2_ derive through independent evolutionary processes. The parameter *t* is the probability that a protein in *G* survives in *G*_1,2_ (proteins are lost with probability 1−*t*); and parameter *s* is the probability that an interaction between proteins, i.e., an edge in *G*, survives in *G*_1,2_ (interactions are lost with probability 1−*s*). With the additional assumption that the ancestor network *G* is an Erdős-Rényi [75] random graph (i.e., a *G*(*n*,*p*) graph with *n* nodes, where each of the $\binom {n}{2}$ possible edges occurs independently with probability 0<*p*<1) this evolutionary model is equivalent to the *G*(*n*,*p*;*t*,*s*) model studied in the literature [42–44].

Under this model, conditions for the success of PGM-based network alignment have been established. In particular, a sharp phase transition in terms of the seed-set size have been shown: If the seed-set size is above some threshold (which depends on the network parameters *n*, *p*, *t*, and *s*), PGM-based alignment can correctly match, with high probability, almost all the node couples by using a purely structural process. Also, from the result of [43], we know that under a similar random bigraph model, the correct alignment maximizes the number of conserved interactions between the two networks. This simple parsimonious evolutionary model provides guarantees for the performance of the PROPER algorithm over random graphs similar to [45]. Note that, in practice, these algorithms are able to successfully align large real-networks, as well as many types of random graphs. In conclusion, it seems that mapping a (small) subset of nodes through a seed-generation step and matching the rest by using only structure of the two graphs works very well under an evolutionary model.

## Conclusion

In this paper, we have introduced a new global pairwise-network alignment algorithm called PROPER. We have compared our algorithm with the state-of-the-art algorithms. We have shown that PROPER outperforms the other algorithms in both accuracy and speed. Also, we have shown that the PROPER algorithm can detect large conserved subnetworks between species.

Our results suggest that network-evolutionary models could be beneficial in designing network alignment algorithms. We believe that, for future work, considering a model that also takes into account gene duplication, network motifs, clustering within networks and modularity of biological networks (e.g., [76]) would increase the accuracy of global network alignments. Finally, to find biological pathways and protein complexes using the PROPER algorithm, the next step would be to design methods that can detect sub-networks as potential pathways or complexes (similar to the method used in [12, 24]).

## Endnotes

^1^ A network motif is a small recurrent connected-subgraph that occurs in PPI and other biological networks significantly more often than in random networks.

^2^ These pathways are Spliceosome. Spliceosome removes introns from a transcribed pre-mRNA, a type of primary transcript.

^3^ Pathways hsa05200 and mmu05200 are in the class cancer Homo sapiens (human).

## Additional files

Additional file 1 IsoBase: experimental results. The results for experiments over Isobase dataset are provided in this appendix. (PDF 269 kb)

Additional file 2 The PROPER algorithm: detailed comparisons. The detailed experimental results for PROPER are provided in this appendix. (PDF 183 kb)

Additional file 3 Pathways: experimental results. The detailed experimental results for aligning biological pathways are provided in this appendix. (PDF 165 kb)

## Abbreviations

BLAST: Basic local-alignment search tool
PGM: Percolation graph matching
PPI: Protein-protein interaction
