# Supplementary material for: PROPER: global protein interaction network alignment through percolation matching
Source: BMC Bioinformatics. 2016 Dec 12;17:527. doi: 10.1186/s12859-016-1395-9 (PMC5153870; doi:10.1186/s12859-016-1395-9)
Supplement: Additional file 1 — IsoBase: experimental results. The results for experiments over Isobase dataset are provided in this appendix. (PDF 269 kb) [file 12859_2016_1395_MOESM1_ESM.pdf]

IsoBase: Experimental Results

Ehsan Kazemi, Hamed Hassani, Matthias Grossglauser and Hassan Pezeshgi Modarres

Isobase is a collection of PPI networks of five major eukaryotic species [4, 1]. This database also contains information about (i) gene ontology (GO) and KEGG categories associated to the proteins, and (ii) functionally related orthologs. In addition, for complementary comparisons, we use the experimentally verified GO terms from [2]. We consider a subset of four species from IsoBase. As the PPI network of M. musculus is very sparse (with average degree 1.867) we omit if from our comparisons. Table 1 represents the name of these species and the number of proteins and interactions in their PPI networks.

Table 1: PPI networks of four eukaryotic species from IsoBase [4].

species	#nodes	#edges	Avg. deg.
C. elegans	2974	4827	3.246
D. melanogaster	7387	24937	6.752
H. sapiens	10296	54654	10.617
S. cerevisiae	5523	82656	29.932

In this appendix, we compare different alignment algorithms. The alignments are done over six pairs of species: ce-dm, ce-hs, ce-sc, dm-hs, dm-sc and hs-sc (for abbreviations and information about the networks refer to Table 1).

Figure 1 compares algorithms based on the average of EC versus GOC score for all the possible 6 pairwise alignments between the species. The complementary result for experimentally verified GO terms is shown in Figure 2.

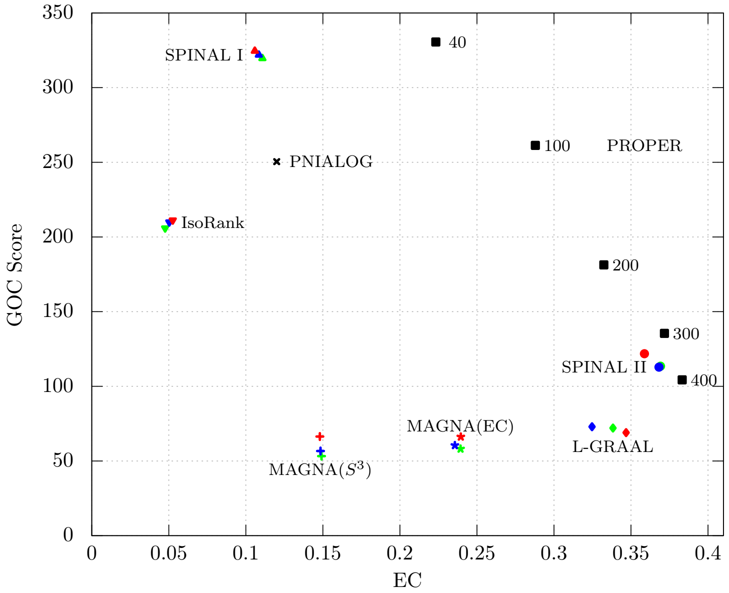

Figure 1: Comparison of different global network-aligners based on the average GO consistency vs. average EC. For the PROPER algorithm, we set $r = 1$ and each point corresponds to a different value of ℓ. The red, blue and green points correspond to the parameters $\alpha = 0.3, 0.5$ and 0.7, respectively.

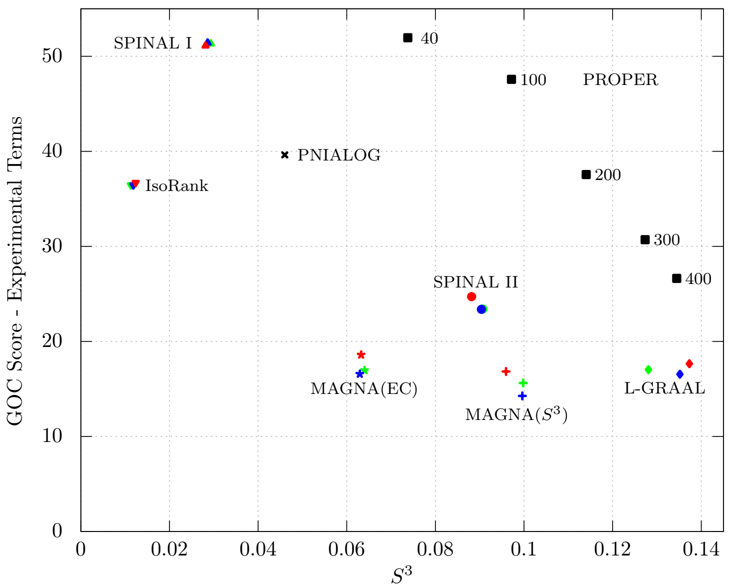

Figure 2: Comparison of different global network-aligners based on the average GO consistency (by considering only experimentally verified GO terms) vs. average S^3. For the PROPER algorithm, we set $r = 1$ and each point corresponds to a different value of ℓ. The red, blue and green points correspond to the parameters $\alpha = 0.3, 0.5$ and 0.7, respectively.

Kyoto Encyclopedia of Genes and Genomes (KEGG) database [3] provides another classification of proteins. We define KEGG consistency similar to the GOC using KEGG categories. Figure 3 compares algorithms based on average KEGG consistency versus average LCSC. We observe that proper finds alignments with high KEGG consistency and LCSC scores.

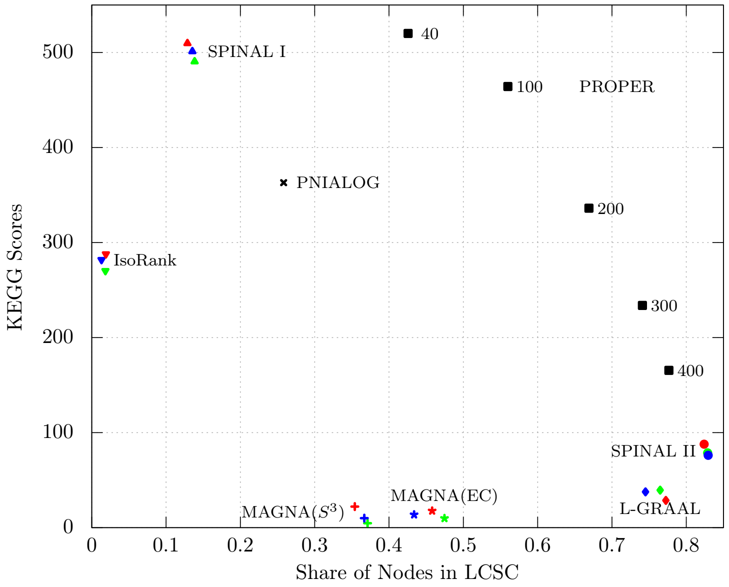

Figure 3: Comparison of different global network-aligners based on the average LCSC vs. average KEGG consistency. For the PROPER algorithm, we set $r = 1$ and each point corresponds to a different value of ℓ. The red, blue and green points correspond to the parameters $\alpha = 0.3, 0.5$ and 0.7, respectively.

Figures 4, 5, 6, 7, 8, 9 and 10 report the detailed results of algorithms for each one of the alignments over all pairs of species.

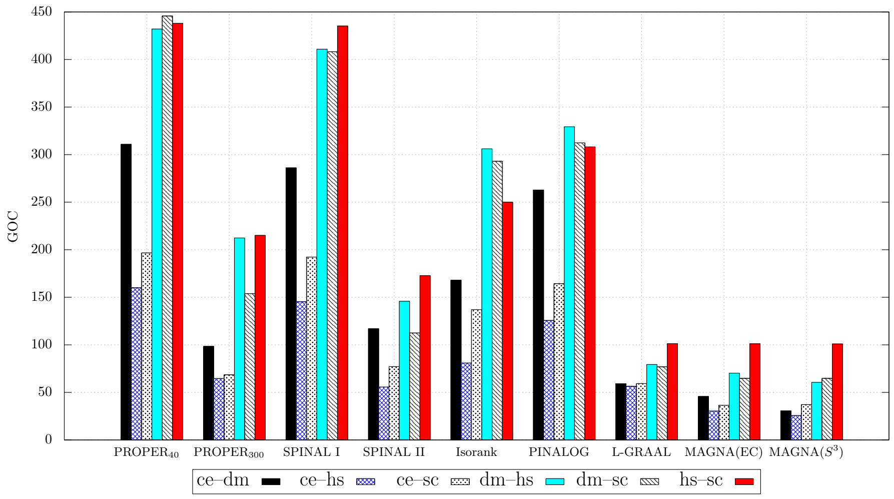

Figure 4: GO Consistency scores for each alignment of the IsoBase PPI-networks. For the PROPER algorithm, we set $r = 1$ and $\ell \in \{40, 300\}$. The parameter α is 0.7.

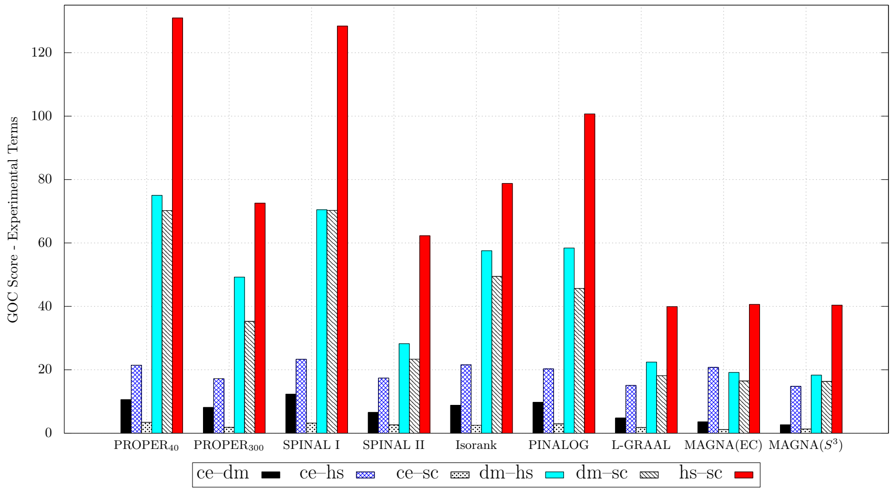

Figure 5: GO Consistency scores (considering only experimental terms) for each alignment of the IsoBase PPI-networks. For the PROPER algorithm, we set $r = 1$ and $\ell \in \{40, 300\}$. The parameter α is 0.7.

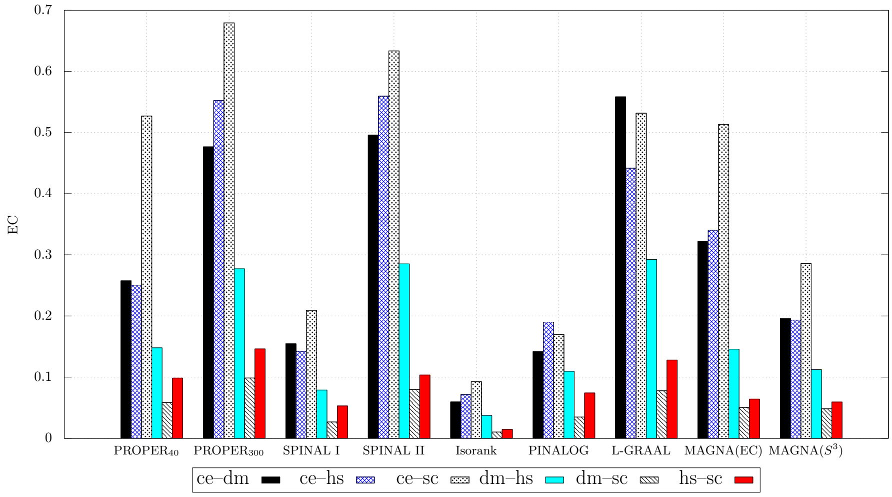

Figure 6: Edge correctness (EC) scores for each of the pairwise alignment for the IsoBase PPI-networks. For the PROPER algorithm, we set $r = 1$ and $\ell \in \{40, 300\}$. The parameter α is 0.7.

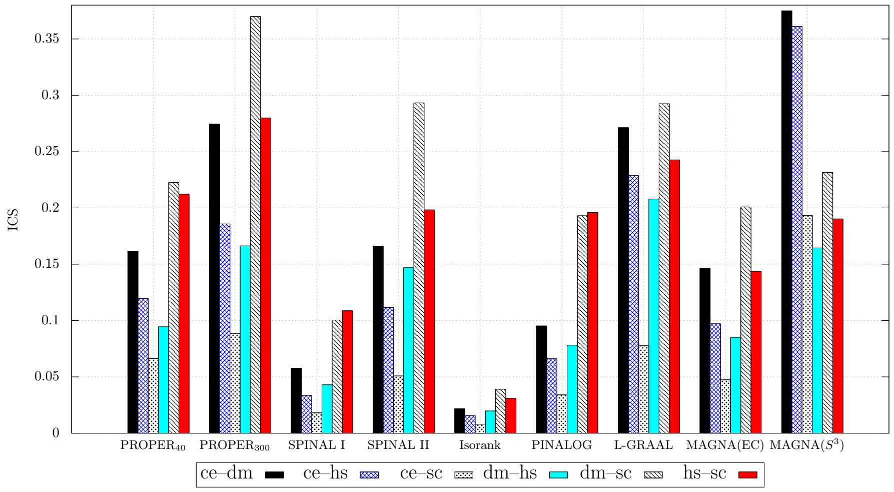

Figure 7: Induced conserved structure (ICS) scores for each of the pairwise alignment for the IsoBase PPI-networks. For the PROPER algorithm, we set $r = 1$ and $\ell \in \{40, 300\}$. The parameter α is 0.7.

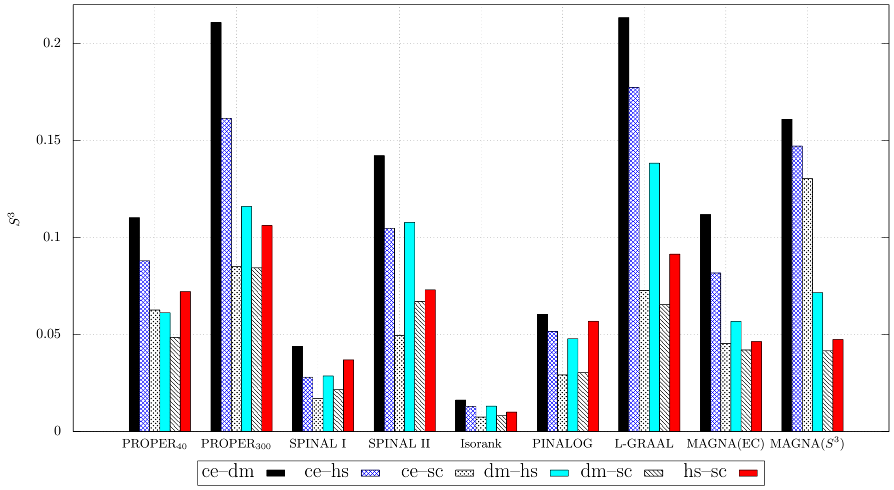

Figure 8: Symmetric substructure score (S^3) scores for each of the pairwise alignment for the IsoBase PPI-networks. For the PROPER algorithm, we set $r = 1$ and $\ell \in \{40, 300\}$. The parameter α is 0.7.

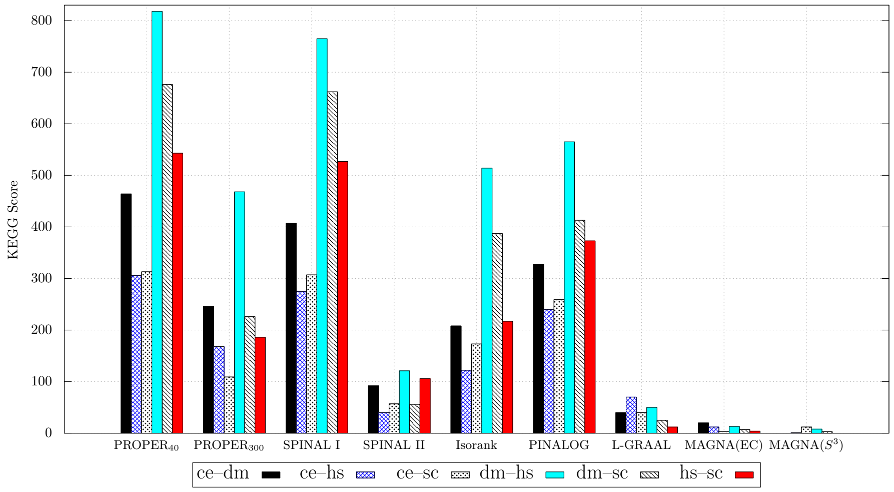

Figure 9: KEGG scores for each alignment of the IsoBase PPI-networks. For the PROPER algorithm, we set $r = 1$ and $\ell \in \{40, 300\}$. The parameter α is 0.7.

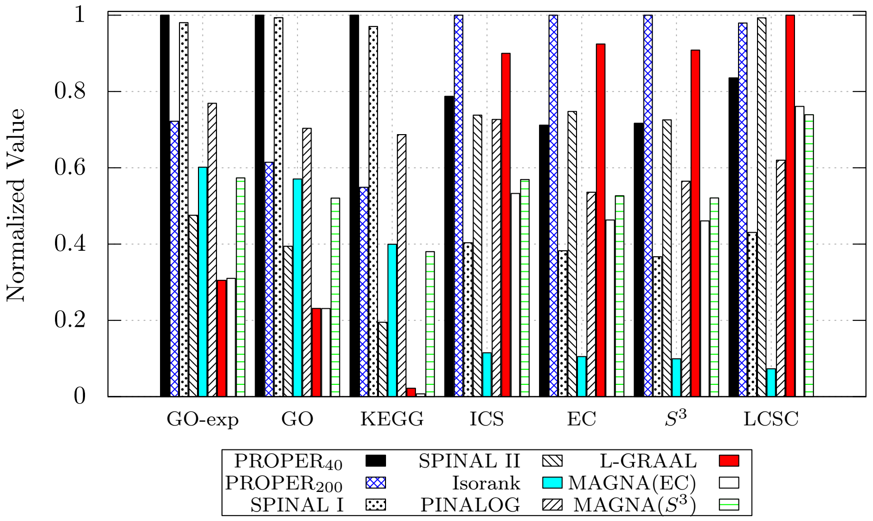

Figure 10: Comparison of different global network-aligners on aligning H. sapiens and S. cerevisiae based on six different measures. For the PROPER algorithm, we set $r = 1$ and $\ell \in \{40, 200\}$. The parameter α is 0.7.

References

[1] IsoBase: A Database of Functionally Related Orthologs. `http://groups.csail.mit.edu/cb/mna/isobase/`. Data acquired on 04 April 2016.

[2] A. E. Aladag and C. Erten. SPINAL: scalable protein interaction network alignment. *Bioinformatics*, 29(7):917–924, 2013.

[3] M. Kanehisa and S. Goto. KEGG: kyoto encyclopedia of genes and genomes. *Nucleic acids research*, 28(1):27–30, 2000.

[4] D. Park, R. Singh, M. Baym, C.-S. Liao, and B. Berger. IsoBase: a database of functionally related proteins across PPI networks. *Nucleic Acids Research*, 39(Database-Issue):295–300, 2011.
