# Supplementary material for: PROPER: global protein interaction network alignment through percolation matching
Source: BMC Bioinformatics. 2016 Dec 12;17:527. doi: 10.1186/s12859-016-1395-9 (PMC5153870; doi:10.1186/s12859-016-1395-9)
Supplement: Additional file 2 — The PROPER algorithm: detailed comparisons. The detailed experimental results for PROPER are provided in this appendix. (PDF 183 kb) [file 12859_2016_1395_MOESM2_ESM.pdf]

The PROPER Algorithm: Detailed Comparisons

Ehsan Kazemi, Hamed Hassani, Matthias Grossglauser and Hassan Pezeshgi Modarres

Table 1: The experimental results for PROPER with different values of $r \in \{1, 2\}$ and $\ell \in \{150, 200, 300, 400, 500, 600\}$. The results are the average of 10 pairwise alignments between species from Table 2.

r, ℓ	$\|\pi\|$	GOC (all)	GOC (exp.)	EC	ICS	S^3	LCSC
1,150	**5521.2**	**1388.562**	**371.460**	0.231	0.218	0.102	3345.7
1,200	5471.4	1284.853	351.285	0.249	0.235	0.112	3610.7
1,300	5432.9	1117.425	321.699	0.273	0.264	0.125	4081.9
1,400	5416.4	999.517	301.476	0.292	0.279	0.135	4397.3
1,500	5347.4	913.508	285.664	0.303	0.285	0.140	4533.4
1,600	5320.5	832.233	271.684	**0.309**	**0.295**	**0.145**	**4669.8**
2,150	3116.1	1224.103	299.481	0.114	0.185	0.060	1375.7
2,200	2900.3	1104.756	275.392	0.122	0.205	0.066	1433.6
2,300	2618.4	920.392	239.140	0.134	0.247	0.075	1566.2
2,400	2408.7	791.523	212.464	0.143	0.269	0.082	1617.8
2,500	2216.1	687.839	191.209	0.147	0.280	0.086	1602.8
2,600	2094.0	603.080	173.923	0.148	0.296	0.089	1596.8

Table 2: PPI networks of five major eukaryotic species from IntAct molecular interaction database [2, 1].

species	#nodes	#edges	Avg. deg.	#pathways
C. elegans	4950	11550	4.67	117
D. melanogaster	8532	26289	6.16	127
H. sapiens	19141	83312	8.71	288
M. musculus	10765	22345	4.15	284
S. cerevisiae	6283	76497	24.35	98

Figure 1 and 2 compare algorithms based on precision over synthetic networks.

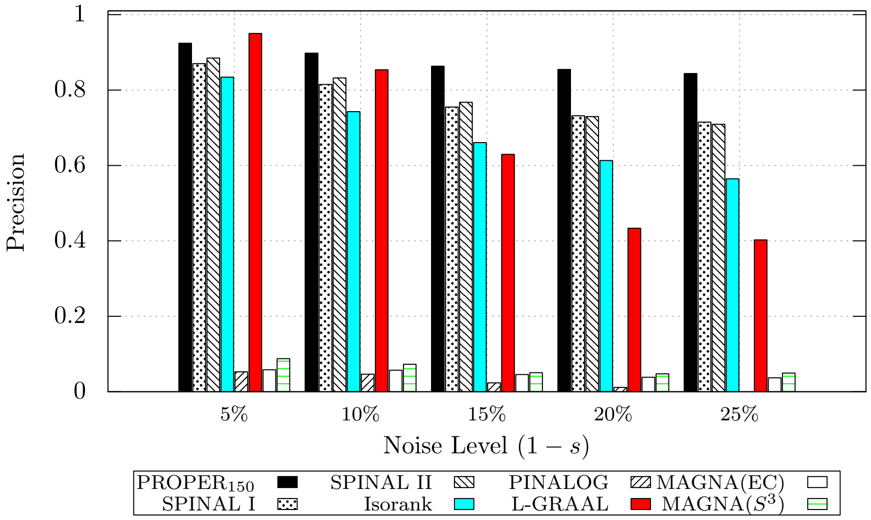

Figure 1: Comparison of different global network-aligners over synthetic networks based on precision. The sequence similarity for 50% of randomly chosen proteins is provided. For the PROPER algorithm, we set $r = 1$ and $\ell = 150$. The parameter α is 0.7.

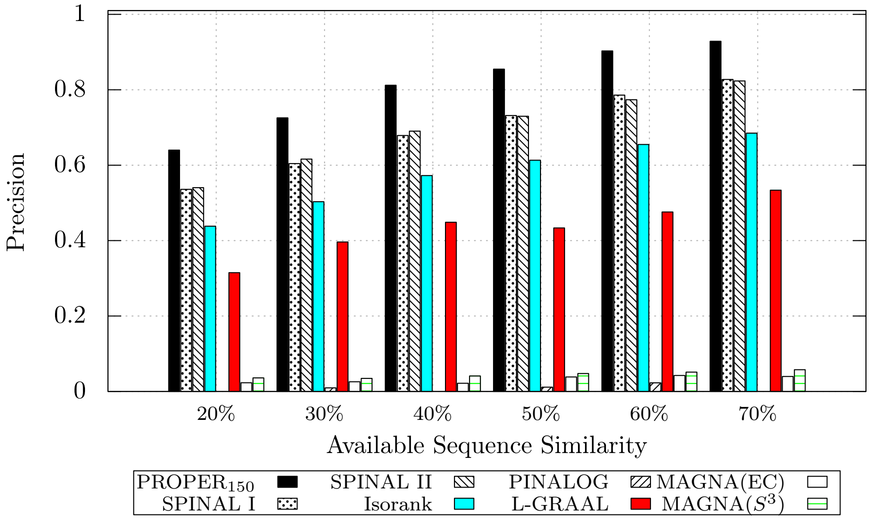

Figure 2: Comparison of different global network-aligners over synthetic networks based on precision. The level of noise is set to $1 - s = 20\%$. For the PROPER algorithm, we set $r = 1$ and $\ell = 150$. The parameter α is 0.7.

References

[1] IntAct: an open source molecular interaction database. http://www.ebi.ac.uk/intact/. Data acquired on 04 April 2016.

[2] H. Hermjakob, L. Montecchi-Palazzi, C. Lewington, S. Mudali, S. Kerrien, S. Orchard, M. Vingron, B. Roechert, P. Roepstorff, A. Valencia, et al. IntAct: an open source molecular interaction database. *Nucleic acids research*, 32(suppl 1):D452–D455, 2004.
