# Supplementary material for: PROPER: global protein interaction network alignment through percolation matching
Source: BMC Bioinformatics. 2016 Dec 12;17:527. doi: 10.1186/s12859-016-1395-9 (PMC5153870; doi:10.1186/s12859-016-1395-9)
Supplement: Additional file 3 — Pathways: experimental results. The detailed experimental results for aligning biological pathways are provided in this appendix. (PDF 165 kb) [file 12859_2016_1395_MOESM3_ESM.pdf]

Pathways: Experimental Results

Ehsan Kazemi, Hamed Hassani, Matthias Grossglauser and Hassan Pezeshgi Modarres

In this appendix, we provide the results of our experiments for aligning biological pathways in more details. The results for each one of the algorithms are reported in Tables 1, 2, 3, 4, 5 and 6.

Table 1: The PROPER algorithm: pathways with at least four conserved edges ($\delta = 4$) in the intersection graph G_0.

species	#PW$_\delta$	#FPW$_\delta$	$\overline{acc_{\pi,\delta}}$	$recall_\pi$
ce-hs	19	8	0.455	0.421
dm-hs	31	12	0.611	0.387
hs-mm	152	128	0.788	0.842
sc-hs	32	22	0.486	0.688
Average	58.5	42.5	0.585	0.584

Table 2: SPINAL I algorithm: pathways with at least four conserved edges ($\delta = 4$) in the intersection graph G_0.

species	#PW$_\delta$	#FPW$_\delta$	$\overline{acc_{\pi,\delta}}$	$recall_\pi$
ce-hs	19	7	0.472	0.368
dm-hs	31	11	0.564	0.355
hs-mm	152	116	0.711	0.763
sc-hs	32	21	0.469	0.656
Average	58.5	38.75	0.554	0.536

Table 3: SPINAL II algorithm: pathways with at least four conserved edges ($\delta = 4$) in the intersection graph G_0.

species	#PW$_\delta$	#FPW$_\delta$	$\overline{acc_{\pi,\delta}}$	$recall_\pi$
ce-hs	19	1	0.301	0.053
dm-hs	31	1	0.081	0.032
hs-mm	152	30	0.261	0.197
sc-hs	32	4	0.247	0.125
Average	58.5	9	0.223	0.102

Table 4: PINALOG algorithm: pathways with at least four conserved edges ($\delta = 4$) in the intersection graph G_0.

species	$\#\mathrm{PW}_\delta$	$\#\mathrm{FPW}_\delta$	$\overline{acc_{\pi,\delta}}$	$recall_\pi$
ce-hs	19	8	0.409	0.421
dm-hs	31	9	0.459	0.290
hs-mm	152	120	0.629	0.789
sc-hs	32	22	0.492	0.687
Average	58.5	39.75	0.497	0.547

Table 5: L-GRAAL algorithm: pathways with at least four conserved edges ($\delta = 4$) in the intersection graph G_0.

species	$\#\mathrm{PW}_\delta$	$\#\mathrm{FPW}_\delta$	$\overline{acc_{\pi,\delta}}$	$recall_\pi$
ce-hs	19	1	0.211	0.053
dm-hs	31	1	0.312	0.032
hs-mm	152	92	0.414	0.605
sc-hs	32	8	0.344	0.250
Average	58.5	25.5	0.320	0.235

Table 6: IsoRank algorithm: pathways with at least four edges conserved ($\delta = 4$) in the intersection graph G_0.

species	$\#\mathrm{PW}_\delta$	$\#\mathrm{FPW}_\delta$	$\overline{acc_{\pi,\delta}}$	$recall_\pi$
ce-hs	19	3	0.341	0.158
dm-hs	31	5	0.442	0.161
hs-mm	152	60	0.407	0.395
sc-hs	32	6	0.234	0.186
Average	58.5	18.5	0.356	0.225

The pathways hsa05200 and mmu05200 are the pathways in the class cancer Homo sapiens (human). The largest connected subgraphs of hsa05200 and mmu05200 pathways in the intersection graph G_0 from all the algorithms are shown in Figures 1, 2, 3, 4, 5 and 6.

Next, we consider another pathway. The largest connected subgraphs of hsa04510 and mmu04510 pathways in the intersection graph G_0 from all the algorithms are shown in Figures 7, 8, 9, 10, 11 and 12. We observe that, again, PROPER returns alignments with a larger portion of connected conserved subgraphs compared to other algorithms.

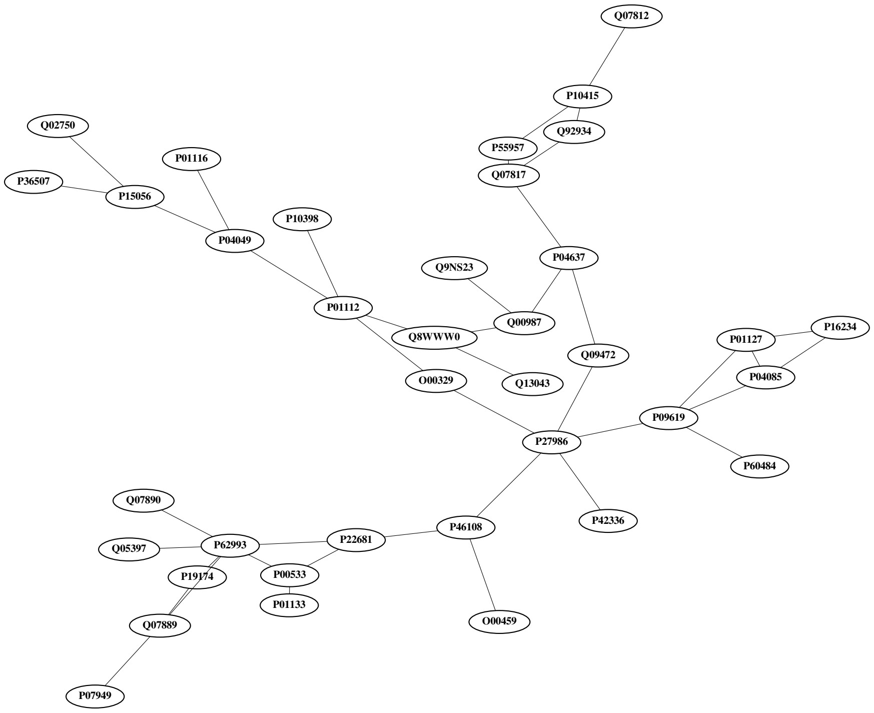

Figure 1: PROPER: pathways hsa05200 and mmu05200. Subgraph is preserved in hs and mm. This connected subgraph of the pathway has 37 nodes and 42 edges.

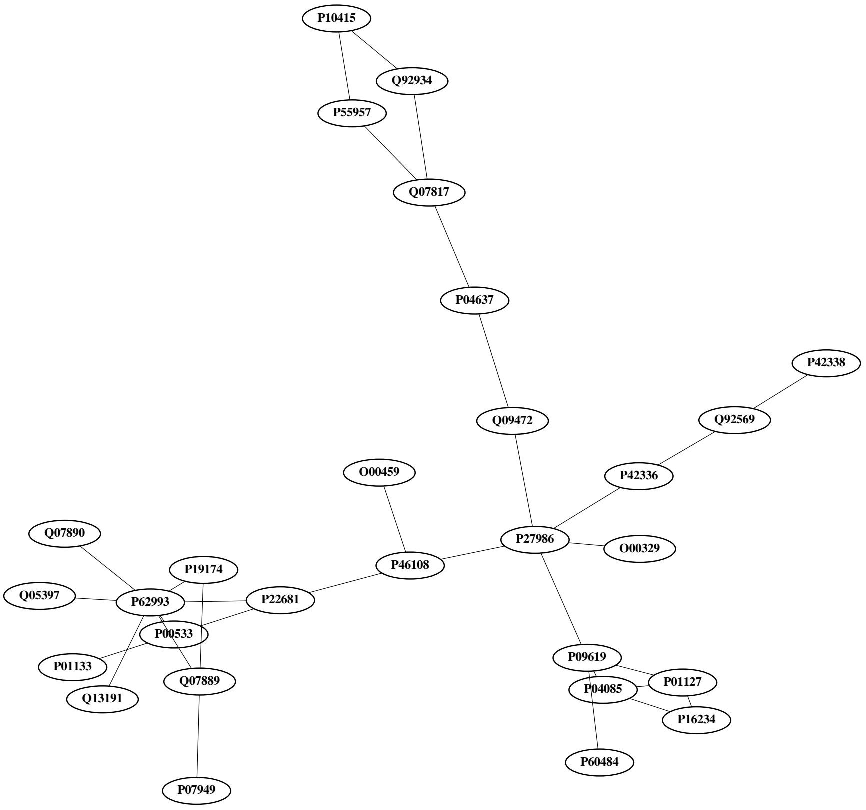

Figure 2: PINALOG: pathways hsa05200 and mmu05200. Subgraph is preserved in hs and mm. This connected subgraph of the pathway has 28 nodes and 32 edges.

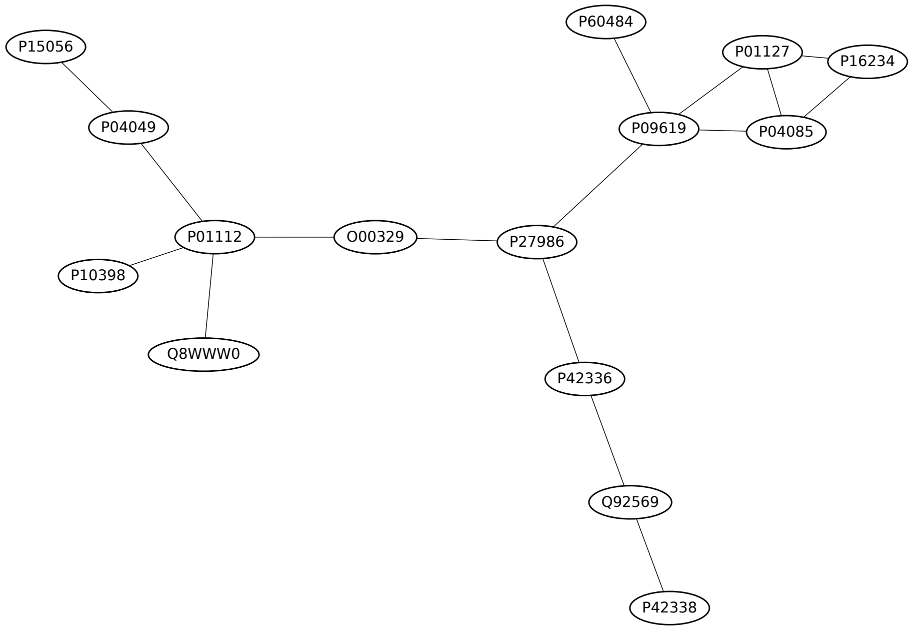

Figure 3: L-GRAAL: pathways hsa05200 and mmu05200. Subgraph is preserved in hs and mm. This connected subgraph of the pathway has 15 nodes and 16 edges.

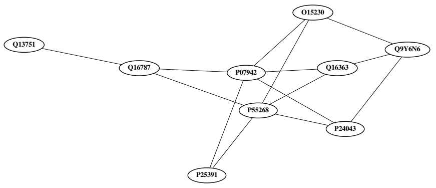

Figure 4: SPINAL I: pathways hsa05200 and mmu05200. Subgraph is preserved in hs and mm. This connected subgraph of the pathway has 9 nodes and 14 edges.

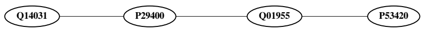

Figure 5: SPINAL II: pathways hsa05200 and mmu05200. Subgraph is preserved in hs and mm. This connected subgraph of the pathway has 4 nodes and 3 edges.

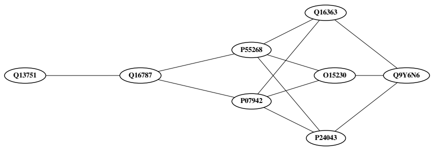

Figure 6: IsoRank: pathways hsa05200 and mmu05200. Subgraph is preserved in hs and mm. This connected subgraph of the pathway has 8 nodes and 12 edges.

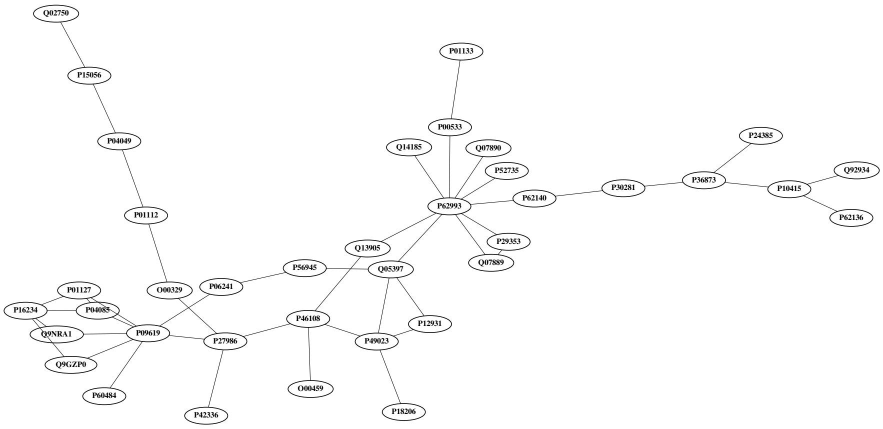

Figure 7: PROPER: pathways hsa04510 and mmu04510. Subgraph is preserved in hs and mm. This connected subgraph of the pathway has 38 nodes and 45 edges.

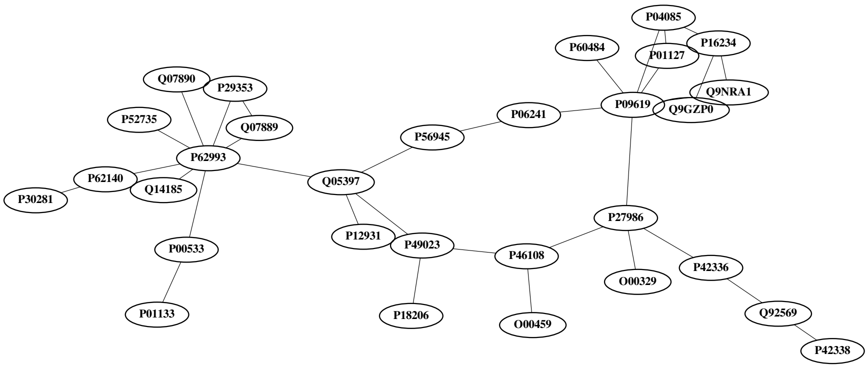

Figure 8: PINALOG: pathways hsa04510 and mmu04510. Subgraph is preserved in hs and mm. This connected subgraph of the pathway has 30 nodes and 36 edges.

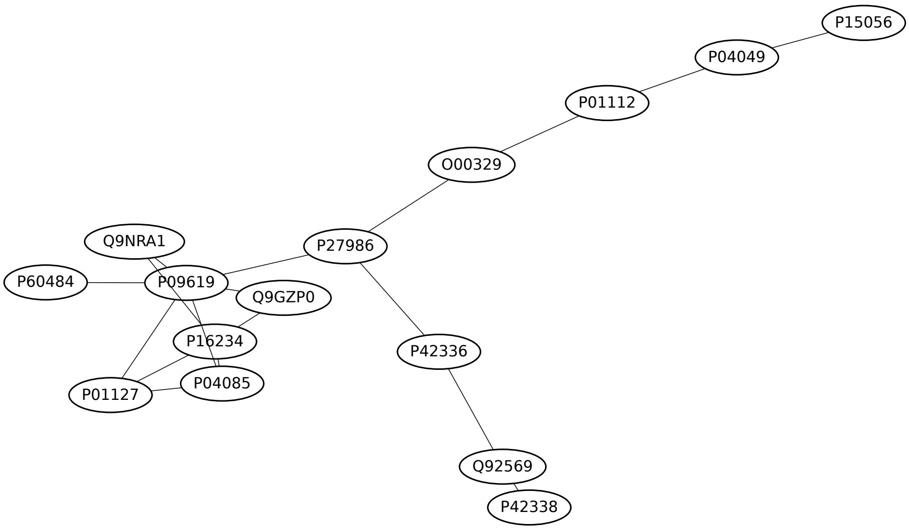

Figure 9: L-GRAAL: pathways hsa04510and mmu04510. Subgraph is preserved in hs and mm. This connected subgraph of the pathway has 15 nodes and 18 edges.

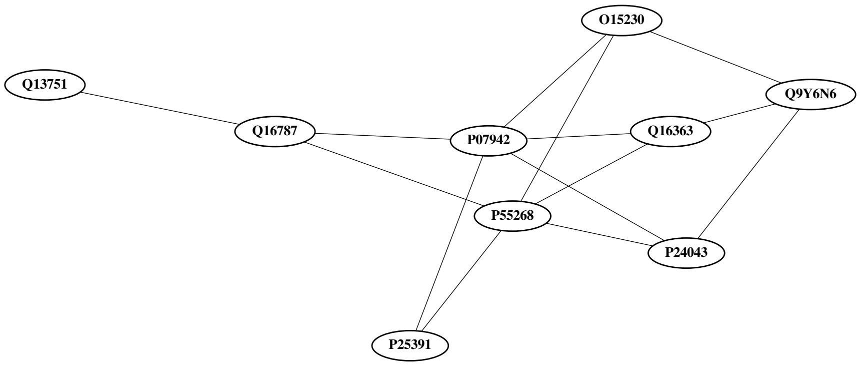

Figure 10: SPINAL I: pathways hsa04510and mmu04510. Subgraph is preserved in hs and mm. This connected subgraph of the pathway has 9 nodes and 14 edges.

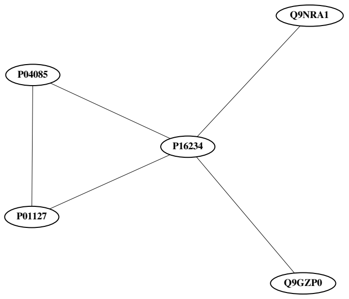

Figure 11: SPINAL II: pathways hsa04510 and mmu04510. Subgraph is preserved in hs and mm. This connected subgraph of the pathway has 5 nodes and 5 edges.

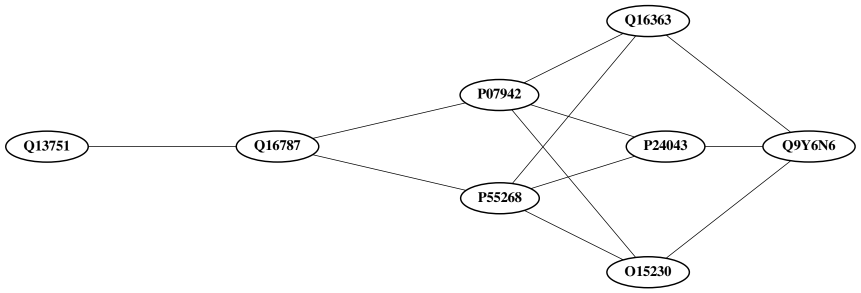

Figure 12: IsoRank: pathways hsa04510 and mmu04510. Subgraph is preserved in hs and mm. This connected subgraph of the pathway has 8 nodes and 12 edges.
